# Found in Translation: Humor and Style Attributes Unveiled by Horton

**DOI:** 10.3390/jemr19040081

**Published:** 2026-07-24

**Authors:** Tanya Beelders, Luna Bergh

**Affiliations:** Computer Science and Informatics, University of the Free State, Bloemfontein 9301, South Africa; berghl@ufs.ac.za

**Keywords:** Dr. Seuss, eye-tracking experiment, prosody, stylometry, syllable stacking

## Abstract

**Background:** The popularity of Dr. Seuss books across generations and languages is worthy of introspective analysis in order to establish how well the translation adheres to the original essence that is the underlying reason for its enduring nature. This article analyses *Horton Hears a Who!* and its Afrikaans translation. **Methods**: A basic stylistic analysis of Dr. Seuss’ *Horton Hears a Who!* was conducted in order to determine whether its rhythmic nature and the meter used in the original text translate well into Afrikaans. Additionally, two extracts were used for an eye-tracking experiment whereby participants were asked to read both so that gaze behavior could be analyzed and compared between Afrikaans and English. **Results**: Eye-tracking results of both passages reveal that readers are comfortably within typical reading behavior and do not experience difficulty when reading the unique style of Dr. Seuss in either language. Stylistic meter does however change the position of elevated fixation durations, but made-up words and the underlying playful tone do not result in difficulty. However, text characteristics, such as length and number of syllables, and word positions, significantly affect gaze behavior. **Conclusions**: It is concluded that the very nature of Dr. Seuss books that makes the stories come alive for children over generations is successfully embodied in the Afrikaans translation with respect to the aspects tested and that both the serious, skillful writing and the more frivolous story-telling tone is present in the Afrikaans target text.

## 1. Introduction

Annually, on 14 February, love is celebrated as Valentine’s Day in many countries. As of 13 years ago, however, love has also been celebrated on this date in South Africa (and 43 other countries) in another respect, as the International Day of Book Giving [1].

Several South African organizations are actively contributing to invoking a life-long love of reading among children via read-aloud projects. For example, the read-aloud Zoë school community project [2] and the efforts of *Nali’bali* [3], who offer free-to-use multilingual stories on their website that can be read aloud. In turn, the *Vriende van Afrikaans* (Friends of Afrikaans) is currently presenting countrywide workshops on storytelling and reading aloud to children and gifting *boektrommels* (book treasure chests) to schools. These projects complement interdisciplinary research on reading intervention via volunteer “grannies” that read stories to rural learners [4].

“It is such love of reading that we need to inspire in communities”; this was one of the motivations behind the 2023 Clarens book festival [5], which was motivated in part as a response to the report that South African grade 4 learners read poorly and without comprehension [6]. The latter shortfall was further exacerbated within the aftermath of COVID-19 [7], and the relative, practical distance of grandparents [8].

Global book sales elsewhere have recently shown an increase in many markets, with children’s books remaining a substantial contributor to print book sales [9]. Indeed, six of the top ten best-selling authors in the UK and seven in Ireland for 2023 were children’s authors [10].

Dr. Seuss books are noticeably well-loved favorites that resonate with a younger audience and even have the power to transcend language barriers and generational gaps. This is clear from the fact that the books have remained in constant publication and circulation since 1937 and have been translated into 50 languages [11].

As the saying goes, *it takes a village to raise a child,* and this is investigated in projects such as that by Stewart and Modiba [4]. In our global village, children can now also draw encouragement from the teenagers and youth known as Generation Z (GenZ, born between 1997 and 2012) in becoming more comfortable around books. Myburg [12] explains that the current reading pattern of GenZ has a positive influence on book sales, given that the Nielsen book data shows that 80% of book purchases between November 2021 and November 2023 were made by GenZ, who as a group have high standards for books and the authority and skill of an author [12]. In addition, libraries have recorded a 71% increase in GenZ visits. Myburg [12] mentions that the oversaturation of noise of digital media has led GenZ to value print books, and that GenZ members prefer a print book, because it relieves strain on the eyes, provides uninterrupted focus on the narrative and enables escape from social media [12].

In our experience, combined with availability, sales and Saturday readings at bookstores, Dr. Seuss books have become engrained in South African culture, remaining popular among “children of all ages” over several decades [13]. The books employ various techniques that appeal to young readers, including a rhythmic or melodic style of writing that repeats throughout the text and often includes “made up words”. Furthermore, the world created in these books gravitates towards the fantastical or whimsical, capturing the attention and creative imagination of youngsters who can easily relate to such worlds of fancy. However, it is these very features that are spurned or ridiculed by some reviewers and educators as being “too frivolous”, effectively discounting these texts as appropriate classroom material [14]. This article argues, however, that books that encourage and foster a love of reading are worthy of further investigation, also because there may be concerns from parents, as well as the linguistic community, about the quality of translated classics chosen for read-aloud settings.

Theoretically, as in some of our other projects, our analysis in this article resides within our cognitive–semiotic model [15,16]. The following six points about cognitive linguistics within the model and our prior research [17] are relevant to the analysis here.

Firstly, our cognitive semiotic model enables an analysis of the value of intersubjective, societal artifacts and rituals. Afrikaans is a Germanic language that developed in Africa. The ritual of reading a story aloud typically leads to a discussion of its meaning and provides a bridge between the heritage of oral storytelling and reading a written story.

Secondly, within the overall model, the branch of cognitive linguistics is a usage-based model, which we view as a continuum:

< context-free grammars–usage-based grammars > pragmatics > sociolinguistics >

With its focus on use, pragmatics, and sociolinguistics, cognitive linguistics is also a communication model. Moreover, in the context of our article, it should be kept in mind that translation is also a form of communication.

The third, related point is that style enjoys rightful status in rigorous linguistic ana-lysis. This means that, in accordance with the continuum above, appropriate style (of a translated book, for instance) would be considered against the grammatical conventions of a linguistic community, the context (for example, children’s stories), and its role and value as artifact in a community. Although we do not remark on further educational implications or test reception of the texts compared to other books, the general societal context sketched so far is in line with our cognitive–semiotic model. This article is largely exploratory in nature, as it posits an innovative method of combining the stylistic properties of text, eye-tracking, and data analysis techniques on a translated work in Afrikaans, which could be used during one or more of the stages of style consideration outlined in this paragraph.

Fourthly, linguistic constructions themselves are context-dependent, form/sound-meaning units. This means that even though we focus on sound aspects here, (conceptual) meaning cannot be disregarded. This needs to be remembered with reference to the Afrikaans title, *Horton Hoor ‘n Hoe!*

Linked to the above points is the notion of embodiment. Langacker [18] explains that “much of the world we live in is mentally and socially constructed. But directly or indirectly, the world we construct and apprehend is grounded in sensory and motor experience. Ultimately, the world we construct is grounded in our experience as creatures with bodies who interact with their surroundings through physical processes involving sensory and motor activity. This is known in cognitive linguistics as *embodiment*”.

Lastly, emotion and language are seen as complex mental systems with knowledge subsystems that interact variously. Our model is thus appropriate for considering cognitive–emotive phenomena such as humor and their relation to style. In this article, the focus is on a verse story; for [19], poetry is more ‘emotional’ than prose.

Our article discusses the verse story *Horton Hears a Who!* and its accompanying Afrikaans translation, *Horton hoor ‘n Hoe!*

The research question is twofold:

1. To what extent does the Afrikaans translation preserve the stylistic features of the source text?
2. How does the reading of these features differ between the source and the target text?

This article considers these research questions with the objective of establishing whether a translated work stays true to the original as a means to harness its essence.

To answer the posed research questions, various techniques will be applied. Firstly, the vocabulary of the book will be analyzed using rudimentary stylometry measures to get a sense of the stylistic characteristics of the texts. Following this, a concise qualitative analysis of the translation and vocabulary use will guide the determination of how well the translation compares to the original. Finally, reading behavior, in terms of gaze movements, will be discussed to determine whether there are differences in the way readers read the original English and the Afrikaans translation and how any differences in the translation affect reading behavior.

### 1.1. Children’s Literature

Keehn et al. [20] emphasize that reading aloud “whets children’s appetite for reading. When we read to young children, they discover the rewards of reading and are motivated to learn to read. In fact, books that are read aloud to young children are frequently the ones they pick up to read themselves when they visit the classroom or school library”.

#### 1.1.1. Humor

Keehn et al. [20] point out that teachers can tell that they are succeeding in motivating a love of reading “when children ask for a book to be read again and again”. Keehn et al. [20] provide five characteristics that may ensure that books are winners in motivating a love of reading. Firstly, “stories with strong, problem-centered plot lines”. Secondly, stories featuring humorous situations. Thirdly, stories with unusual formats and distinctive illustrations. Young children furthermore relish books that invite movement. Lastly, books that are multicultural in nature.

Humor can be defined as “intentional verbal and nonverbal messages which elicit laughter, chuckling, and other forms of spontaneous behavior taken to mean pleasure, delight, and/or surprise in the targeted receiver” (Booth-Butterfield and Booth-Butterfield 1991 in [21]). The story analyzed in our article includes at least two types of humor in a setting of ‘dissent’ [21]; namely, the sour mother kangaroo laughing scornfully and both kangaroos calling Horton a fool for hearing and attending to a soft voice, and Horton keeping his calm while performing the ironic impossibility of a huge animal helping an almost invisible human.

One does not typically think of humor in sacred texts; yet this paradox might lead to some thought-provoking principles and examples of human folly. Ryken et al. [22] categorize types of humor in the Bible and assert that, “arranged on a continuum that ranges from the least intellectual (slapstick comedy) to the most intellectual (irony and wordplay), we can conclude that the humor of the Bible tends toward the subtle” [22]. They emphasize that the “humor of the Bible is not of the rollicking type but the subtle and intellectual type for which the term *wit* is often an accurate designation” [22].

Acrostics represent a term that is associated with poetry in the Bible. In Biblical Hebrew, acrostic psalms or poems of passages typically refer to “poetic passages that use the Hebrew *alphabet* as its structure” [23]. Such Hebrew poems use the letters of the alphabet in their set order “to begin a new line strophe, unit or paragraph” [20]. There are at least five functions of acrostics: namely, mnemonics for memory; enumeration; showing completeness, wholeness or totality of what is to be said on the topic; aesthetics; capturing cognitive aspects, such that faith is not only emotional, but also rational; and visual and aural effects [23]. Although the use of acrostics influences the impact of a poem, translators have been urged not to attempt replicating this skill in the target text, given the near impossibility of doing so [23]. As far as we know, playfulness has not been associated with acrostics before, yet this link is evident in the verse story discussed in our article.

The makeup of Dr. Seuss books eschewed by some educators and reviewers is, at second glance, not as frivolous as it seems [24]. While the underlying tenor of the book remains light-hearted, whimsical and full of fun, the tone belies the meticulous and skillful writing of the author [14]. For example, the words that are habitually made up by the author might initially be disregarded as gobbledygook, but in fact are coined to assist with the meter or have strong German roots [25]. The books invariably offer social commentary of a deeper nature (for example, greed, over-commercialization) or address a moral theme such as responsibility and respect for others—which is evident in *Horton Hears a Who!*

#### 1.1.2. Stylistic Properties of Children’s Books

As was mentioned earlier, rhyme and meter are often explicitly employed to draw children into the story and keep them engaged and interested. Rhyme, rhythm and meter are the three features in our analysis that resort under prosody—which is the art of versification, “the rules governing the technique of verse-making” [19]. Rhyme can be described as “the repetition of a particular sound”, often at the end of the lines of poetry [19]. Rhythm, in turn, is the “harmony produced by the regular repetition of a regular beat or accent. All good poetry must have rhythm, but rhyme is not essential” [19]. Rhythm is marked by beats and when the beats follow one another in a regular sequence, the arrangement of those beats is termed *meter*, the recurrence of beats dividing the line into what are termed *feet*. A *foot* is made up of two or three syllables, one accented and one unaccented, or one accented and two unaccented [19].

Since children prefer repetitive or perpetual rhyming to a rhythm that changes [26], this is another factor that illustrates how cleverly the books were crafted. Dr. Seuss books, including *Horton Hears a Who!*, frequently employ anapestic (penta-)meter. A three-syllable unit with the accent at the end is called an anapest, and four such units or feet constitute a tetrameter. Below is an example paragraph from *Horton* showing the meter:

On the FIFteenth of MAY, in the JUNgle of NOOL,

In the HEAT of the DAY, in the COOL of the POOL,

He was SPLASHing… enJOYing the JUNgle’s great JOYS…

When HORton the ELephant HEARD a small NOISE.

This in itself was innovative during that publishing period, as Gorell [27] explains: in English, the main, the most characteristic meter is the five-foot iambic [one unaccented followed by an accented syllable] which, because of “its suitability to the stresses of our tongue, has gradually come to dominate almost all our poems of length and weighty purpose”.

All these features make the popularity and importance of Dr. Seuss well worth investigating. They also raise the question of how well translated versions convey these same principles. This paper will discuss coinages, meter and nuances found in the Afrikaans version of *Horton* with respect to analyzing style.

### 1.2. Stylometry

The word *style* is generally used in various contexts in the cultural life of societies, but a general, societal language convention is that correctness relates to grammar, whereas appropriateness of choice relates to style [28]. Stylometry, in turn, is the study of text characteristics, based on the principle that every author develops their own linguistic style [29]. Stylometry can be used to verify authorship, identify an author or characterize an author based on the fact that individuals have a unique writing fingerprint or linguistic style [29]. This is manifested in the writer’s vocabulary, in particular, choosing certain words over others [30], as well as the four types of stylometric entities listed below [29]:

Lexical features such as the number and length of sentences and words;
Syntactic characteristics such as punctuation and the use of function words (articles, prepositions, pronouns);
Structural features such as the organization of sentences within paragraphs and of paragraphs within the text;
Content-specific features that can be used in interaction situations.

There are many examples of studies in stylometry applied to translations [31,32]. The stylometric parameters of sentence complexity, as demonstrated by sentence length, type/token ratio, average word frequency and number of rare words [31], word frequencies, concordance and cluster analysis [32] were considered relevant to translation studies [31]. For the purpose of this paper, since it is concerned with translation and how well the essence of the original text is preserved between two languages that have similar syntactic structures, only lexical features ((i) above) will be analyzed, in conjunction with punctuation, articles and the sound elements of rhyme, rhythm, and meter. Consequently, the analysis tends towards a stylistic comparison rather than a full-blown stylometry analysis.

#### Translation

An in-depth discussion of translation theory lies beyond the scope of our article. Instead, our translation discussion is generally based on the combined insights of [33] (in relation to our cognitive–semiotic model, and the translation of poetry, sacred texts and humor) and [34] (knighted for her translations of French literature into Afrikaans). Morgan [34] views translation as an art and a science, yet feels that literary translation is a music exercise, rather than a linguistic exercise, given the importance of melody, tone, rhythm and sound production. In turn, Baker [33] emphasizes the balance between accuracy and naturalness in translation.

### 1.3. Gaze Movements While Reading

Much research has been conducted on gaze movements while reading various formats and languages (for example, [35,36,37]). When reading, a human’s gaze alternates between fixations and saccades (the latter is not of interest to this study). Fixations are periods of time during which the eye is held relatively still. A fixation is used to “see” an object or read a word. When reading English, these fixations typically last between 200 ms and 250 ms [38], but there is large variability between fixation durations on individual words as cognitive processing (often at the end of a sentence) and reading difficulty both cause an increase in fixation duration [39]. On average, there are 1.2 fixations per word, with short function words often being skipped [39]. The number of words per fixation decreases as the complexity of the text increases or the reading skill of the reader decreases [39]. However, a skilled reader has less fixations when reading a simple text—for instance, an adult reading a children’s book [39]. Regressions are fixations that fall onto a word that has been read or viewed before. These are used to cement and understand something that has been read; to verify something that was read before; or increase when the reader is experiencing difficulty with the text being read.

The location, length, and number of fixations are of interest in reading research, as these give an indication of the difficulty the reader is experiencing while reading portions of the text. These studies, often considered benchmarks, were however conducted using English L1 readers who read texts in English. Different languages yield different reading behavior as a result.

In terms of Afrikaans reading, prior research has shown that there is a difference in gaze behavior between first language (L1) and second language (L2) readers. In particular, L2 readers have both more and longer fixations than L1 readers [40]. Afrikaans readers, on average, fixated once per syllable in a word and exhibited a visual span of between 3.72 and 5.13 characters. Fixation durations while reading an easy Afrikaans text were, on average, 313 ms in length, while this increased to an average of 331 ms for a more difficult text [40]. In terms of typical English reading behavior, the findings of [40] show that the visual span is less for typical English reading; hence, one would also expect that the number of fixations is more in Afrikaans than English reading [40]. Fixation durations were also longer than for a typical English reader reading in L1.

## 2. Materials and Methods

### 2.1. Hardware

**Figure 1:**
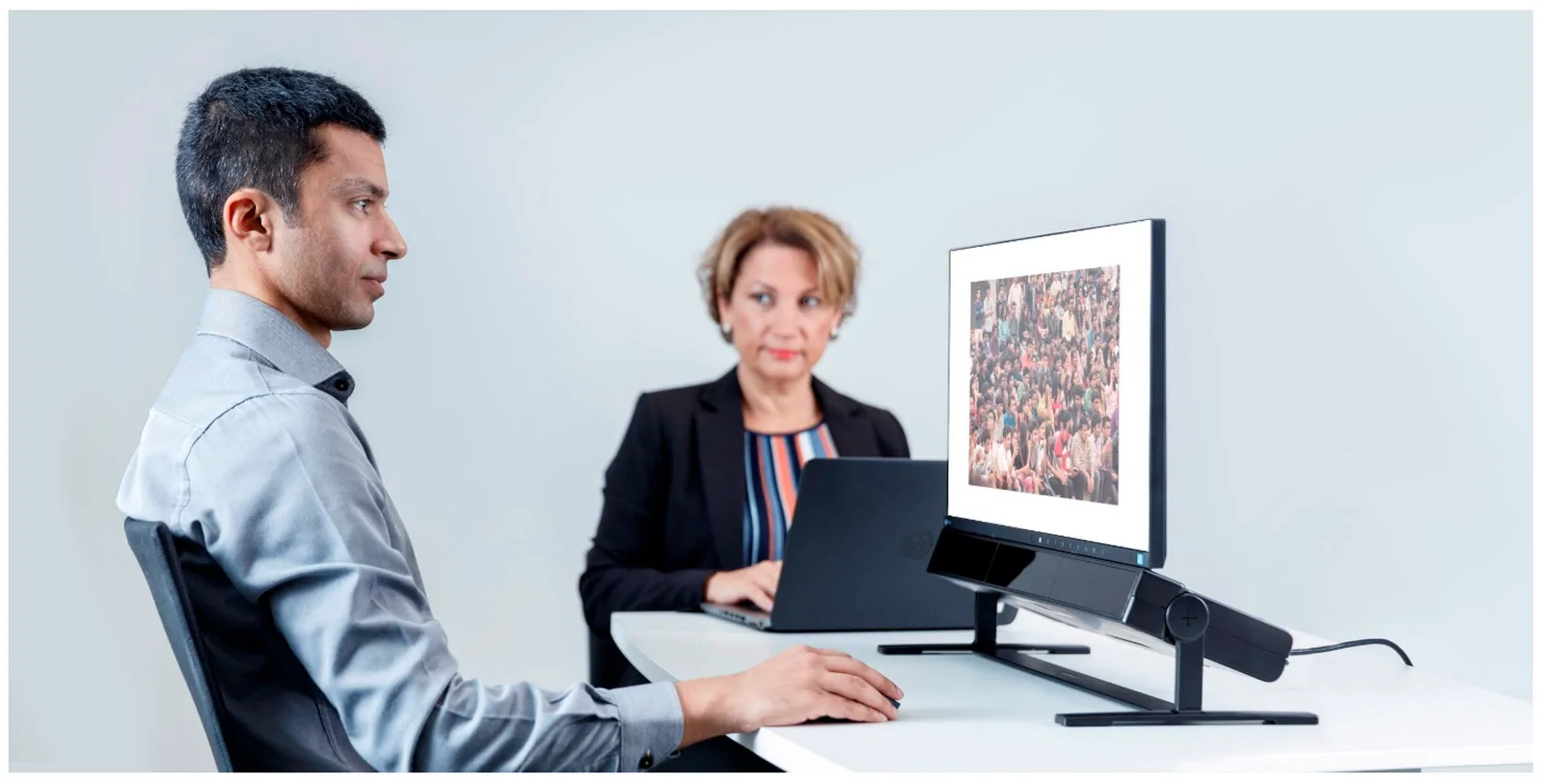
The Tobii Spectrum eye-tracker in a typical test setup environment [42].

An eye-tracker is a piece of hardware that can capture eye movements while a stimulus is presented to a participant [41]. For this study, the Tobii Spectrum (Figure 1) eye-tracker was used to capture gaze while participants read a digital text-only version of *Horton*. The Spectrum is a remote eye-tracker that is mounted below a normal computer screen. This allows free head movement and a natural setting whereby the participant feels as though they are simply reading on a computer screen (see Figure 1 for a typical setup). Participants were seated approximately 60 cm from the screen. Data was captured at a rate of 600 Hz. Tobii Pro Lab software version 1.181.37603 (×64) was used to extract fixations and saccades. The Tobii-I-VT fixation filter was used to analyze and identify fixations. For these purposes, the standard settings for fixation identification were used, namely minimum fixation duration of 60 ms and a velocity threshold of 30 degrees/second. Fixations were extracted using the reading data metric export feature of Tobii Pro Lab. Only metrics with null or no metric data were discarded.

### 2.2. Participants

Participants were staff and students of the university where the study was conducted who volunteered to participate. All participants had normal or corrected-to-normal vision and were fluent in English and Afrikaans as both speakers and readers. The fluency of the participants was confirmed verbally when inviting them to participate—the criteria being that they must be comfortable and fluent reading in both English and Afrikaans. Consequently, and together with the fact that the texts represented children’s stories, it was not considered vital to distinguish between L1 and L2 Afrikaans speakers. The text was slightly enlarged and widely spaced to ensure accuracy of data capturing, and it was assumed that participants would indicate whether they were experiencing difficulty reading or seeing the text; hence, no formal vision test was administered. Participants were informed about the procedure and reason for the study, and all signed a consent form prior to participation. In total, there were 37 participants who read both passages. While this is a small sample, eye-tracking studies often have smaller samples due to the intensive nature of capturing the data on an individual basis, which is a time-consuming exercise. Members of the respective genders and generational groups were represented. Including students enabled the participation of GenZ, an influential generation mentioned earlier in the Introduction. Since the study was paired and no influencing factors were anticipated, no participant demographics were collected beforehand.

Our research resides in the niche of reading stories aloud to children. Both staff and students may fit into the categories of parents, caregivers and au pairs who read stories to children. In this way, the current study complements research based on children’s perceptions of books. In view of anonymity and confidentiality, no distinction was drawn between staff and student participants.

The study was approved for ethical clearance (anonymized).

### 2.3. Stimuli

The stimulus presented was an extract from a Dr. Seuss book entitled *Horton Hears a Who!* [43]. The same extract, from the official, published Afrikaans versions, entitled *Horton Hoor ‘n Hoe!*, was also presented [44]. Dr Seuss was chosen as the included author for the study due to the popularity of his books, the tone of his writing and the availability of translated versions.

Dr. Seuss books are color-coded according to the reading level. *Horton Hears a Who!* is classified as a yellow-back, making it more suitable for older (end of grade 3) fluent children to read on their own. As was pointed out earlier in the Introduction, local learners in this age category have been mentioned repeatedly in the media for poor reading skills. As a result, it would not be appropriate to expose such learners even further via eye-tracking. In addition, for a valid, comparable study, the same anonymous learners from the other surveys would also need to be participants in our study. Including children in our experiment would also not foster love of reading in the current context. Notwithstanding, a longitudinal (see [4]), experimental translation reception study (see [45]) that includes learners in this category as participants (see [46]) could be worthwhile in future.

Since *Horton Hears a Who*! *is* a yellow-back book, it is also one of the longer Dr. Seuss books. Hence, it was decided to only present an extract—yet the entire story—of the book to minimize the testing time, as tests were conducted during work and lecture hours. Therefore, for each of the English and Afrikaans texts, the first 21 pages were shown. The first pages were used so that the reader starts the story and can immerse themselves rather than having to start reading in the middle and being unable to understand the context or orient themselves in the story. The extracts end on page 21 where the *Who* on the dust speck talks to Horton and Horton responds. The last sentence of the extract reads as follows: ‘And Horton called back to the Mayor of the town, “You’re safe now. Don’t worry. I won’t let you down.”’

Furthermore, given our focus on reading-aloud stories, adults were sought as participants. Reading aloud is already an essential component of the translation process and target product, in that the translator needs to hear how characters, dialog and phrases sound in the target language [34]. The countrywide presentation of storytelling and reading-aloud-to-children workshops, also mentioned in our Introduction, confirms that not all adults are confident readers or story performers, even though they may read fluently by and for themselves. For this reason, the readability of texts was also considered in our eye-tracking experiment. Since the participants were all adults, a more advanced Dr. Seuss book was chosen. Three factors especially maximize the complexity of reading this advanced text; namely, the genre of poetry, new words and syllable-rich phrases.

Because the study is only concerned with reading and the translated words, all images were removed and only the text was presented. This partly emanates from research observations that “most of them [listening learners] did not know their sounds. Not only the English sounds. They did not know their Zulu sounds” [4] (p. 162). This sound-form focus also enriches our research on visual aspects [47] and recent publications [36] that combine text and images. Together, these pave the way for further audiovisual research on humor, style and translation reception [44]. Positive reception of translated classics cannot be taken for granted [48]; yet, given the entrenched popularity of Dr. Seuss books—as expounded earlier in the Introduction—our demarcated study posits the convergence of stylometry, eye-tracking and a concise qualitative analysis as a method in such an overall systematic evaluation.

The presentation of texts was counter-balanced—that is, the order was alternated between subsequent participants to ensure that no other factors, such as tiredness or order of presentation, influenced reading behavior unduly. Therefore, to control for potential order effects, a Latin square approach was adopted whereby every other participant was presented the English text first, and alternating participants were presented with the Afrikaans text first, proceeding sequentially in this fashion. Participants were allowed to read at their own pace and silently. No comprehension was tested afterwards, given that only adults participated and that meaning-making would be more appropriate in a read-aloud-to-children ritual setting, in accordance with our cognitive–semiotic model.

## 3. Results

### 3.1. Stylistic Characteristics

Note that the stylistic features discussed here were calculated only for the passages that were read, not the whole book of stories, and are aimed at facilitating a stylistic comparison. As previously mentioned, lexical, structural and syntactic characteristics were selected as appropriate for this study.

The characteristics of each text are tabulated in Table 1.

This clearly shows that sentences in the Afrikaans text tend to be much longer than the English text. This is contrary to typical Afrikaans spoken and written language, where sentences are inclined to be shorter [28]. This could be unique to the translation of Dr. Seuss texts, due to the nature of the underlying meter and unconventional use of punctuation in the original text.

Paragraphs were identified using the spacing on the page. At the level of graphology, the Afrikaans paragraphs mimic the original text. Dr. Seuss books often do not use conventional punctuation or paragraph breaks. This is the case with *Horton,* where each page contains a paragraph, but line breaks are used to maintain the meter and do not follow typical English line breaks. In addition, the end of a line might often appear to the reader to be the end of a thought or idea, even though it may not be concluded with a punctuation mark.

Table 2 contains a few paragraphs from the *Horton* text, as well as the same extract from the Afrikaans text. To illustrate the unusual punctuation, notice line 1 in the English extract. Upon reading line 1, it could easily be interpreted as a single thought/idea, but the line does not end with a full stop or exclamation point. Instead, the sentence continues on the next line, which then ends with a full stop. This is generally known as enjambement or caesura in poetry. The same phenomenon is seen on lines 4 and 5, as well as lines 6 and 7. In terms of paragraph demarcation, lines 1–5 are a single page and, for the purposes of the paper, are seen as a paragraph. Punctuation marks were used at the end of a sentence.

Regarding the Afrikaans extract, the line count is not identical to the original source text, but the meter is mimicked very closely. The sentiment is also related very closely, even though not necessarily on identical lines. For example, the referent on line 11 “too small for an elephant’s eye”, is found on line 7 in the Afrikaans “Vir my olifantsoog is dié spikkel te klein”.

The descriptive language is well translated; the second paragraph concentrates on conveying how small this person is, even smaller than a speck of dust—far too small for an elephant to see! In Afrikaans, this is articulated as “byna onsigbaar en nietiglik fyn”. However, the language used in both the English and Afrikaans versions is much richer and far more descriptive than the loose translation of “almost invisible and tiny”. In the same way, “some sort of creature…” on line 10 is described in Afrikaans as “fyn spikkeltjie wesentjies”, which creates the illusion of a fine, tiny speck of a character. In this way, the description invokes a realistic and imaginative depiction of a creature tinier than a speck of dust and it uses the original wording of speck later in the Afrikaans text.

**Figure 2:**
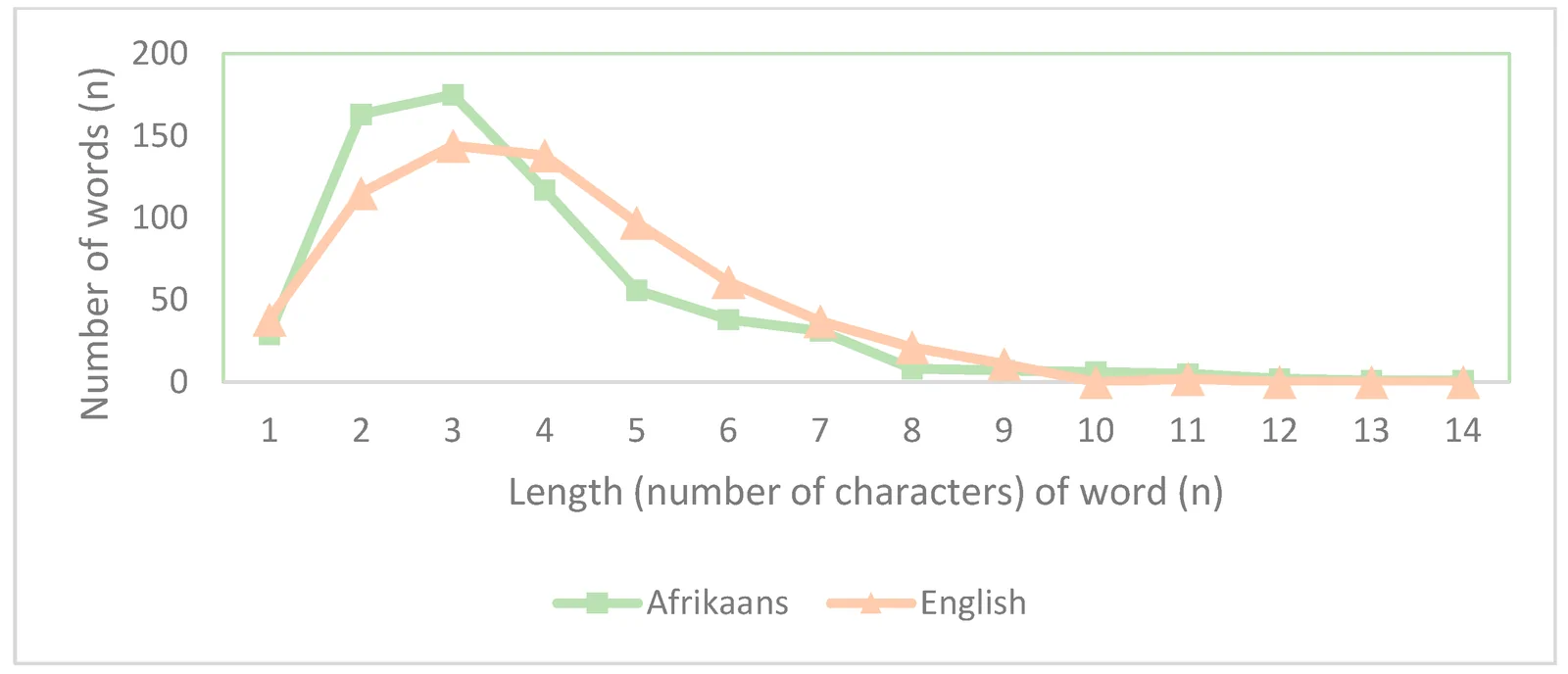
Number of words broken down by word length.

Figure 2 shows a breakdown of the number of words (excluding punctuation marks) of each length for the two passages.

As can be seen, the majority of the words have shorter lengths for both languages, with minimal words of longer length in either language. The Afrikaans passage contained more shorter words than the English passage, while the English passage contained more words of 4–9 characters in length. The difference is not substantial and thus it indicates that, in terms of the word length, the pieces are very similar.

Based on the premise that paragraphs of the translated text contain the same idea and construct as the English text, paragraphs can be grouped as individual segments that could be paired for further analysis. Therefore, for each paragraph, the number of words and average word length (including punctuation but excluding spaces) were calculated and a Pearson correlation was conducted to determine how closely they correlated to one another.

The word count per paragraph is very closely correlated (r = 0.97, *p* < 0.001), meaning that as the length of the English version grows in terms of the number of words, so does the Afrikaans text. Therefore, the content and narrative flow of the two extracts are closely aligned.

The average word length correlation was very close to zero (r = −0.13, *p* = 0.7); therefore, there was no meaningful relationship between the average length of words in English and Afrikaans.

### 3.2. Lexical Features and Meter as Style Elements

In general, diminutives abound in the variant of Afrikaans spoken in South Africa. Our text reveals that diminutives, as well as neologisms and new compounds (such as *krieselklankie*), seem to be typical of Afrikaans usage in this verse story. Lexical features such as rhythmic syllable stacking in relation to meter appear to be major style elements in the target text, as further linked in the Discussion below. Two differences between prose and poetry mentioned in Fletcher and Sceales [19] are noteworthy here. Firstly, “the language of poetry is usually more picturesque”; some of the devices used for this purpose are figures of speech, such as personification, and “poetic devices designed to create special effects”, such as alliteration (“the repetition at very short intervals of the same sound” as in “So Horton stopped splashing”, “tree tops” and “black-bottomed bird” in our source text), assonance (“the repetition of the same vowel sound in two or more different words but without the repetition of the same consonant sounds”, as in “So he plucked up the clover and hustled away” (line 54 in our source text)) and onomatopoeia (“the use of a word suggesting the sound that it represents”, such as “splashing” and “Humpf!” in our source text). Secondly, “poetry is usually briefer and more condensed than prose. It therefore needs to be read with greater concentration”.

### 3.3. Gaze Metrics for the Whole Passage

The nature of Dr. Seuss is complex in that it appears to be simple, yet the underlying message is serious and mature. The exceedingly clever use of meter makes it appealing to younger readers, whilst at the same time conveying an important social message and using complex stylistic elements. The use of the meter throughout, as well as the beguiling complexity hidden within the simplicity, is of interest for reading research, and thus the study investigated whether the nature of these passages influenced reading behavior in any way. Additionally, the translation of the passage adheres to the true Dr. Seuss style; hence, the reading behavior between the languages will also be investigated.

The eye-tracking results reported in this paper were selected to complement the tone and nature of the preceding and forthcoming analysis and discussion, which are more focused on the stylistics and contemplation of the translation of Dr. Seuss. Hence, for the purposes of this paper, statistical analysis of the gaze metrics is included, but this is complemented with qualitative analysis and drilled-down visualizations and discussions about particular phenomena that were identified as potentially relevant for a translation study.

RQ2 asks how eye movements are influenced by translated works. To this end, the following hypotheses are stated:

**H_0,1_:**  *There is no difference in the length of fixations when reading Dr. Seuss in English or Afrikaans.*

**H_0,2_:**  *There is no difference between the number of fixations when reading Dr. Seuss in English or Afrikaans.*

**H_0,3_:**  *There is no difference in total fixation (active reading time) when reading Dr. Seuss in English or Afrikaans.*

**H_0,5_:**  *There is no difference between the first-pass first-fixation duration when reading Dr. Seuss in English or Afrikaans.*

**H_0,5_:**  *There is no difference in the length of visits when reading Dr. Seuss in English or Afrikaans.*

**H_0,6_:**  *There is no difference in the number of visits when reading Dr. Seuss in English or Afrikaans.*

The mean fixation duration for both languages (Table 3) is well within the typical reading range for English L1 reading, as per Rayner [38]. Since the presented stimulus is a children’s book, it was expected that neither version would present an overtly difficult challenge to participants. In terms of the first-pass duration, it appears that the initial reading of a word does not cause an increase in the length of the fixation required, as these are, on average, well within the typical reading fixation duration. Mean visit durations are somewhat elevated, and larger than typical reading for both languages, but since this is composed of multiple fixations, and the mean number of visits is more than one fixation per word, this is not necessarily indicative of reading difficulty, but does indicate that regressions were present as words were visited, on average, more than once.

Since all participants read both English and Afrikaans, the data was paired, and a repeated-measures analysis was conducted. Due to the small sample size and the fact that the data was not normal, the nonparametric Wilcoxon signed-rank test was used for pair analysis. Table 4 contains the results of the statistical analysis for the eye-tracking metrics.

There is no significant difference between the fixation durations when reading English or Afrikaans. This is contrary to previous findings for adult Afrikaans readers [40]. Therefore, this could be attributed to the nature of the passage, as it is a Dr. Seuss book, and a children’s book, which might be easier to read.

Mean reading time, viewed in terms of total fixations, was not significantly longer in either English or Afrikaans. Therefore, reading passages that are similar in terms of lexical stylistic characteristics but differ in language does not require more time.

First-pass mean fixation duration was not significantly longer in either language. First-pass durations could indicate the time taken to read a word upon first glance. Longer times would mean that there is difficulty in immediately reading the word. However, the non-significant difference in this metric indicates that neither language poses significant difficulty in immediately being able to read a word.

In terms of the number of fixations, there was a significant difference between the number of fixations per word; hence, on average, the participants read with more fixations per word in Afrikaans than in English. This correlates with previous studies [40] where L2 readers exhibited more fixations when reading.

Neither the number of visits nor the duration of the visits was significantly different between the languages. Repeated visits to a word indicate that readers are experiencing difficulty in reading or understanding what is being read. Since no significant difference was detected, it can be concluded that neither language presented a significantly more difficult piece to read.

Therefore, only H_0,2_ could be rejected as there are more fixations required for reading Afrikaans words. However, the fixations were not necessarily longer; hence, participants were not experiencing difficulty but required more fixations to read an Afrikaans word, perhaps due to the (un)familiarity of the word or even the length. Since this was the only significant eye-tracking metric, the analysis proceeded by investigating the nature of the words.

### 3.4. Word-Level Observations

This section will provide anecdotal observations of the word characteristics and stylistic features as they relate to gaze behavior. The subsequent section will elucidate these aspects further by reporting on statistical tests.

The 20 Afrikaans and English words with the longest mean fixation durations were extracted for the sake of inspection. Interestingly, and contrary to prior findings [39], the words with the longest mean fixation duration for both languages were all short, and the majority of them were at the start of a sentence or a new line.

Afrikaans words such as *dit*, *elke*, *ek*, *hy* and English words such as *I*, *for*, *in* and *as* elicited the longest fixation durations. In terms of reading behavior, the typical mean fixation duration when reading English was well exceeded, but the average number of fixations, however, was comfortably below the typical 1.2 fixations. This indicates that the participants were not necessarily experiencing difficulty when reading. The longer fixations could therefore be a sign of increased concentration or understanding occurring. Since this occurs at the start of a line, it would seem that the structural makeup of the text could affect this behavior.

The 20 Afrikaans (Table 5) and English (Table 6) words with the greatest number of fixations were extracted for observation.

*Krieselklankie* employs stacked syllables and, like *olifantsoog* and *olifantplig*, is not a standard Afrikaans word. *Piepkleine* and *doodbang* are intensive forms of *small* and *scared*, respectively. These, together with words such as *nietiglik* and *wraggieswaar*, are rich in meaning and depth, using a single compound word to convey much meaning.

In terms of the English words, words such as *humpf*, *speck-voice* and *Who-ville* are not English words but rather are quintessentially made-up Dr. Seuss words. Some of the English words in Table 6 employ onomatopoeia and this could mean readers required more fixations to assimilate the word that represents the sound, not that they were experiencing difficulty.

For both languages, the mean number of fixations per word is well above typical reading behavior, but contrary to the words with the longest fixations, these fixations are all within the typical reading fixation length. This indicates that there were many fixations, but that they were not overly long. The words with more fixations are all longer, more complex, or difficult words than those with longer mean fixation durations. They are all also multisyllabic words.

Both texts contained unfamiliar words, but it could be that the Afrikaans words were seemingly more unfamiliar than the English words and this could account for the increased number of fixations. The majority of the words were longer and comprised multiple syllables. This could explain the increased number of fixations since it has been noted that Afrikaans readers fixate at least once per syllable when reading in Afrikaans.

The metrics concerning the number and duration of fixations, as they relate to the length and number of syllables, are summarized in Table 7 and Table 8.

Neither the mean duration for Afrikaans nor that for English highlights a trend or appears to correlate with the length of the word. Since participants were fluent adult readers of both English and Afrikaans, this is not overly surprising. However, as the length of the word increases, the number of fixations also increases for English and Afrikaans. Hence, the word length might not be a significant predictor of fixation duration, but it can possibly be a predictor for the number of fixations.

Fixation durations are seemingly not elevated as the number of syllables increases, as they remain comfortably within typical reading metrics, but they do appear to get longer as the number of syllables increases. Therefore, there is a definite increase in the number of fixations as the syllable count increases.

Since these are merely averages, the actual data must be analyzed in order to draw concrete conclusions.

The observations discussed in this section are anecdotal in nature and presented to complement the stylistic exploratory gist of the paper. The following section will present a more robust analysis of stylistic features as they influence gaze behavior while reading.

### 3.5. Gaze Metrics for Word Characteristics

Previous sections have noted that the structure of sentences and lines does not adhere to conventional text in order to preserve the meter and style of Dr. Seuss. Additionally, interesting observations regarding gaze behavior and words were noted and must be investigated further.

Therefore, for each word read, the fixation duration and number of fixations were determined. Together with this, the lexical and structural features of the word were identified, namely whether the word was the first or last word in the sentence and whether it was the first or last word on the line.

A multiple linear regression analysis was then conducted to examine the influence of word characteristics and text position on duration and number of fixations. The overall regression model indicated that the factors significantly influenced fixation duration, F(7, 24,805) = 294.6, *p* < 0.001, and number of fixations, F(7, 34,805) = 479.8, *p* < 0.001. However, the model explained a small overall proportion of the variance in fixation duration (r-square = 0.056) and number of fixations (r square = 0.09), reflecting the highly variable nature of individual reading behavior.

The individual t-statistics for each factor are presented in Table 9 for fixation duration and Table 10 for the number of fixations.

Both word length and syllable count significantly increase the fixation duration, as well as the number of fixations on each word. Words at the start of a sentence or line do not significantly affect the fixation duration, but the end of a line and sentence, text position reduces the length of the fixations. This explains the observed behavior that the longest fixations are at the start of a word or sentence, but only because the end of a sentence or line exhibits significantly lower fixations than the rest of the words.

Fixation durations on Afrikaans words were significantly longer than those on English words.

Regarding the number of fixations, word length and the number of syllables caused a significant increase in the number of fixations, while the first word on a line, the first word in a sentence, and the last word in a line resulted in significantly fewer fixations on the word. Afrikaans words also required significantly more fixations than English words.

## 4. Discussion

This paper is largely exploratory in nature, as it uses an innovative method that combines the stylistic properties of text, eye-tracking and empirical data analysis techniques on a translated work in Afrikaans.

The eye-tracking results reveal that fluent adult readers of Dr. Seuss exhibit similar reading patterns in the two languages, with mean fixations between 200 ms and 250 ms, which align with prior findings [38]. In the same way, the number of fixations was found to be similar to previous reading studies [38] that used English L1 adult readers. Only the number of fixations was significantly different when conducting a paired analysis for English and Afrikaans reading. When reading in Afrikaans, significantly more fixations are used, but they are not significantly longer and words do not require significantly longer first-pass reading. Hence, readers’ behavior did not change significantly between the languages when paired for analysis.

However, contrary to previous studies that found longer fixations at the end of sentences due to cognitive processing, this study revealed long fixations at the start of new lines or sentences. This could be attributed to sense-making on behalf of the participants, the influencing effects of the structural and metrical format of the text, or the finding that the end of a line and sentence required significantly shorter fixations. In general, Afrikaans words required both longer and more fixations to read than English words.

Interestingly, a definite correlation was detected in both languages between syllables and fixations, whereby the more syllables there were, the more and longer fixations were made. This indicates that readers require more fixations to read multi-syllable words. However, the fixations did not necessarily overlay the syllables.

In *Horton Hears a Who!* anapestic meter is variously employed, especially by alternating between tetrameter and pentameter. The Afrikaans translation is more systematic in that tetrameter is used throughout. A clear example is found in the second line of the second stanza:

In die LUG, in die POEL, in die WOUD, op die GROND …

(In the SKY, in the POOL, in the WOOD, on the GROUND …)

What is also more systematic in the Afrikaans translation is the use of a rhyme scheme consisting of rhyming couplets using all 26 letters of the alphabet in their set order. If ever there were doubts about rhyming potential in Afrikaans verse, *Horton hoor ‘n Hoe!* puts that to rest—even around the double negative. Acrostics is thus used playfully in the source text (where the end of a line coincides with the imagined beginning of a line in Biblical Hebrew, which is read from right to left) and, remarkably, also in the Afrikaans translation. In comparing the texts, we enjoy both the “conscious art of the highly skilled craftsman” and the seemingly “careless ease of the supreme artist” [27].

In terms of prosody, the Afrikaans translation is thus true to the source text. What needs to be kept in mind is that when the source text was produced, the Oxford English Dictionary definition of poetry was “composition in verse or metrical language” [27]. In the design of both texts, the style is anchored in the meter. By keeping to the source text’s meter, the Afrikaans translation ensures credibility and authenticity. The choice of meter also answers to the question raised above about long fixations on short words at the beginning of new lines in both languages. Gorell [27] explains that the English sentence does not normally begin with a stressed syllable, but with a weak one, an “a” or a “the,” for preference, and the writer wishing to invert the stress meets with an initial difficulty.

Each of the lines in the source and translation encloses a complete thought. On the one hand, this retains the pattern and goals of acrostics. Because of the choice of anapestic meter, each line furthermore ends on a stressed syllable—which, together with the paired rhyming and, most probably, in combination with line position, punctuation and visual effects, sentence-initial processing, wrap-up processes, sentence boundaries, line breaks and capitalization patterns, enforces a fixation on the focus position of the new thought or idea at the beginning of each next line. Our overall results show how sound elements are foregrounded and read similarly in both languages, yet the plot and conceptual meaning flow with these enforced initial line fixations.

The Afrikaans text presents a literary translation; in other words, it is not a direct, word-for-word translation throughout. This choice enables a flowing rhythm and systematic metrical pattern through stacking syllables in word coinages and alliteration (for example, *klein krieselklankie wat krap*; *swaai toe sy slurp met ‘n swiep deur die lug*), as well as words and word order that may be more natural in terms of Afrikaans usage and conventions. In light of our discussion, both verse texts abide by Samuel Taylor Coleridge’s “homely definition of poetry” [27] as “the best words in the best order”—and in accordance with our cognitive–semiotic model, within the creativity and style delineated by linguistic conventions; in the context of a children’s story; and in these cases, already sanctioned as timeless poetry (regarding the essentials of sincerity, clarity and simplicity [27]) for enjoyment in the respective reading communities.

From the exposition in the preceding sections, it follows that translations of popular authors’ work reveal how the attributes of the target language emerge in an effort to capture something of the source language text. In our analysis of meaning and form, the playfulness of sound repetition and long coinages in Afrikaans are unveiled.

Through exploratory stylistic analysis, it was discovered that both texts display the five characteristics of winners in motivating a love of reading [15]. The h-alliteration in the title of the Afrikaans translation takes the second characteristic, humor, to another level of originality in that *Hoe* echoes *Who* and so sets the tone for the playfulness to follow. It also unveils an attribute intrinsic to Afrikaans; namely, humor is a response to a challenge (here, the translation of an artful text), as processed, construed, and embodied by the translator and manifested in the target text. This attribute may be unique to Afrikaans and represents a challenge for translations into other languages.

## 5. Limitations

The sample size used in the study was relatively small and very specific, thereby limiting the generalizability of the study. While eye-tracking studies often have smaller samples due to the individual and intensive, time-consuming nature of data collection, ideally, samples should be larger.

Secondly, the sample consisted of adult readers, while the focus is on children’s books. As mentioned, this was deliberately done to focus on adults as read-aloud partners. Furthermore, this study was exploratory in nature, aiming to investigate the validity of the integration of eye-tracking, stylistics and linguistics before expanding the study to include children.

Thirdly, only an extract—albeit one full story—from the book was chosen for inclusion to reduce the amount of time required by participants. Further studies should consider including the entire book as children’s books are inherently shorter in length. Only using an extract again limits the generalizability of the results.

Further studies of this nature should include short comprehension tests, as well as questionnaires designed to elicit enjoyment, appreciation, and understanding of the humor elements, to ensure that participants are actively engaging with and understanding the text, instead of relying solely on the integrity of the participants. Robust evaluation of prior exposure to the texts should also be captured prior to the reading intervention.

Related to this, for future data collection, it is advisable that the fluency and skill level of participants in all languages being tested be objectively measured to ensure that the language being read is fully understood and the participant is at an acceptable level regarding the language skill set. Additionally, more detailed demographics should be collected to promote the replicability of the study.

## 6. Conclusions

The translation of the Dr. Seuss verse story in our study provides an easy-to-read text, and its unique nature, both in terms of vocabulary and meter, do not cause reading difficulty for adults who might read it aloud to young children. The stylistic nature of the text does, however, change reading behavior significantly, but again does not appear to cause reading difficulty, merely changing the way in which readers pause to process, understand and make sense of the text being read.

The translation stays true to the original text through the use of descriptive, rich text, as well as the use of punctuation, line breaks, and paragraph breaks to maintain rhythm and meter.

It is concluded, based on the stylistic analysis conducted and the tested aspects, that much of the very nature of Dr. Seuss’s books—which makes the stories come alive for children over generations—is successfully embodied in the Afrikaans translation, through the employment of various stylistic components, and that both the serious, skillful writing and the more frivolous storytelling tone are present in the Afrikaans verse story. The preservation of humor and the overall effectiveness of the translation are inferred from the stylistic analyses and adult eye-tracking data rather than direct measures of reader reception. As much as playful humor and style are intertwined in our study, it follows that humor evaluation needs to be unveiled via questionnaires, interviews or read-aloud settings and conversations, in accordance with our cognitive–semiotic model.

## Figures and Tables

**Table 1:** Stylistic features of English and Afrikaans *Horton*.

Stylistic Feature	English Measure	Afrikaans Measure
Number of words	666	636
Average characters per word	4.4	4.0
Longest word length	11	14
Number of paragraphs	11	11
Number of sentences	75	45
Average sentences per paragraph	6.5	4.09
Words per sentence	9.2	13.8

**Table 2:** Extracts from English and Afrikaans *Horton* texts.

English extract	Then he heard it again! Just a very faint yelp As if some tiny person were calling for help. “I’ll help you,” said Horton. “But who are you? Where?” He looked and he looked. He could see nothing there But a small speck of dust blowing past through the air. “I say!” murmured Horton. “I’ve never heard tell Of a small speck of dust that is able to yell. So you know what I think?…Why, I think that there must Be someone on top of that small speck of dust! Some sort of a creature of very small size, Too small to be seen by an elephant’s eyes… “…some poor little person who’s shaking with fear That he’ll blow in the pool! He has no way to steer!
Afrikaans extract	Hy is skoon verwonderd, hy is skoon oorstelp, Want iewers, ja, êrens roep iemand: “o, help!” “Ek help graag,” sê Horton, “maar sê my waar’s jy?” Hy soek, maar daar vlieg net ‘n spikkel verby. Hy skud toe sy kop en hy sê toe: “O nee! Dit is mos onmoontlik dat spikkels kan skree! Vir my olifantsoog is dié spikkel te klein; Ja, byna onsigbaar en nietiglik fyn. Maar kyk ek dalk nader, sal ek miskien oplet Dat hierdie fyn spikkeltjie wesentjies op het. Nou gaan ek vir seker dié wesentjies red, Al is dit dalk moeilik, al is dit geen pret.

**Table 3:** Eye-tracking metrics for English and Afrikaans in *Horton.*

Eye-Tracking Metric	Metric for English Words	Metric for Afrikaans Words
Mean fixation duration (whole passage)	205.7 ms	208.9 ms
Mean fixation duration per word	208.7 ms	208.8 ms
Mean total fixation time	2.76 min	2.58 min
Mean first-pass first-fixation duration (only non-zero first pass)	206.3 ms	206.2 ms
Mean number of fixations per word	1.1	1.3
Mean visit duration	258.7 ms	272.9 ms
Mean number of visits	1.3	1.3

**Table 4:** Paired analysis results for eye-tracking metrics.

	W	Z	*p*	CI	r Effect Size	Median
Mean fixation duration	311	−0.6	0.472	95% [−28.60, 40.90],	0.115 Small	1.550
Mean total fixation time	271	−1.21	0.182	95% [−6.05 × 10^4^, 1.19 × 10^5^]	0.229 Small	9346.00
Mean first-pass first fixation duration (including zero first pass)	350	−0.02	0.983	95% [−38.81, 49.91]	−0.004 Negligible	−0.02
Mean number of fixations	155	−2.2	0.019 *	95% [−0.30, 0.90]	0.447 Medium	0.10
Mean visit duration	236	−1.52	0.101	95% [−43.08, 4.14]	0.291 Small	9.14
Mean number of visits	233	−1.09	0.221	95% [−0.14, 0.25]	0.217 Small	0.02

* Significant *p*-value for a 0.05 α-value.

**Table 5:** Afrikaans words with the highest mean number of fixations.

Afrikaans Words	Mean Number of Fixations (n)	Mean Fixation Duration (ms)
krieselklankie	4.4	219.4
kangaroe-ma	4.3	213.0
klawerplant	4.3	221.6
Burgermeester	3.8	197.4
wraggieswaar	3.6	204.9
olifantsoog	3.6	192.7
Wesentjies	3.2	222.8
Nietiglik	3.1	264.8
olifantsplig	2.9	211.4
Kangaroes	2.9	198.2
Piepkleine	2.9	200.1
Hoor	2.8	208.6
Hoe-ville	2.8	211.2
Grootste	2.8	176.3
Onsigbaar	2.8	202.3
Strakkies	2.8	209.9
verwonderd	2.7	195.1
Doodbang	2.7	222.7
onmoontlik	2.6	184.7
Waars	2.5	218.6

**Table 6:** English words with the highest mean number of fixations.

English Words	Mean Number of Fixations (n)	Mean Fixation Duration (ms)
speck-voice	4.2	193.1
Horton	3.4	233.3
Who-ville	3.4	206.9
Hears	3.3	213.3
Humpfed	2.9	236.9
Fifteenth	2.6	171.8
Murmured	2.5	213.2
Humpf	2.4	198.1
Splashing	2.3	195.2
Looked	2.2	179.3
Terrible	2.2	198.1
Buildings	2.1	201.0
Never	2.1	222.1
Someone	2.1	194.3
Whats	2.1	193.7
wonderfully	2.1	186.7
Thought	2.1	206.3
Gently	2.1	199.4
Jungles	2.1	201.8
Persons	2.1	198.7

**Table 7:** Fixation characteristics according to word lengths.

Number of Characters in Word (No Punctuation)	Mean Duration of Fixations (ms)		Mean Number of Fixations (n)	
	Afrikaans	English	Afrikaans	English
1	200.4	215.6	0.5	0.4
2	216.4	211.6	0.8	0.7
3	210.2	209.2	1.1	0.9
4	209.1	206.1	1.4	1.2
5	202.0	204.6	1.5	1.5
6	206.8	201.6	1.8	1.5
7	202.2	206.1	1.9	1.6
8	205.7	198.4	2.1	1.8
9	216.0	195.2	2.6	1.9
10	203.9		2.2	
11	209.2	191.0	3.3	3.2
12	207.8		3.3	
13	197.4		3.8	
14	219.4		4.4	

**Table 8:** Fixation characteristics according to the number of syllables.

Number of Syllables in Word	Mean Fixation Duration (ms)		Mean Number of Fixations per Syllable (n)	
	Afrikaans	English	Afrikaans	English
1	207.1	186.7	1.1	1.0
2	212.1	195.0	1.8	1.6
3	205.7	199.6	2.6	1.7
4	209.7	207.7	3.6	2.1

**Table 9:** Regression analysis of fixation durations, word characteristics and text position.

Factor	Coefficient	Standard Error.	t	*p*
Word length	25.32	0.96	26.40	<0.001
Syllables	15.77	3.70	4.27	<0.001
First word in sentence	13.09	7.75	1.69	0.091
First word on line	11.24	6.70	1.68	0.093
Last word in sentence	−43.48	5.99	−7.26	<0.001
Last word in line	−30.50	5.53	−5.52	<0.001
Language	31.39	2.72	11.53	<0.001

**Table 10:** Regression analysis of the number of fixations, word characteristics and text position.

Factor	Coefficient	Standard Error	t	*p*
Word length	0.13	0.004	34.22	<0.001
Syllables	0.07	0.01	4.58	<0.001
First word in sentence	0.04	0.03	1.23	0.218
First word online	−0.14	0.03	−5.10	<0.001
Last word in sentence	−0.21	0.02	−8.77	<0.001
Last word in line	−0.13	0.02	−5.87	<0.001
Language	0.13	0.01	12.12	<0.001

## Data Availability

Data is available from the authors on request due to ethical restrictions.
